# High-fat diet accelerate hepatic fatty acids synthesis in offspring male rats induced by perinatal exposure to nonylphenol

**DOI:** 10.1186/s40360-021-00492-z

**Published:** 2021-04-27

**Authors:** Hongyu Zhang, Chengguang Song, Rong Yan, Hongbo Cai, Yi Zhou, Xiaoyu Ke

**Affiliations:** 1 School of Biological and Pharmaceutical Engineering, Wuhan Polytechnic University, Wuhan, China; 2 Department of Emergency, Tongji Hosptial of Tongji Medical College of Huazhong University of Science and Technology, 1095 Jiefang Avenue, Wuhan, 430030 China

**Keywords:** Nonylphenol, High-fat-diet, Perinatal exposure, Fatty acid, Transgeneration

## Abstract

**Background:**

Low dose of NP exposure can alter adipose tissue formation, and the intake of high-fat diet (HFD) can also lead to the fatty liver disease. We investigated the combined effect of NP and HFD on the first offspring of rats, and whether this effect can be passed to the next generation and the possible mechanisms involved.

**Methods:**

Pregnant rats had access to be treated with 5 μg/kg/day NP and normal diet. The first generation rats were given normal diet and HFD on postnatal day 21, respectively. Then the second generation rats started to only receive normal diet without NP or HFD. Body weight, organ coefficient of liver tissues, lipid profile, biochemical indexes and the expression of genes involved in lipid metabolism, as well as liver histopathology were investigated in male offspring of rats.

**Results:**

NP and HFD interaction had significant effect on the birth weight, body weight and liver tissue organ coefficient of first generation male rats. And HFD aggravated abnormal lipid metabolism, even abnormal liver function and liver histopathological damage of first generation male rats produced by the NP. And this effect can be passed on to the second generation rats. HFD also accelerated the mRNA level of fatty acid synthesis genes such as *Lpl*, *Fas*, *Srebp-1* and *Ppar-γ* of first generation rats induced by perinatal exposure to NP, even passed on to the second generation of male rats. NP and HFD resulted in synergistical decrease of the protein expression level of ERα in liver tissue in F2 male rats.

**Conclusion:**

HFD and NP synergistically accelerated synthesis of fatty acids in liver of male offspring rats through reducing the expression of ERα, which induced abnormal lipid metabolism, abnormal liver function and hepatic steatosis. Moreover, all of these damage passed on to the next generation rats.

**Supplementary Information:**

The online version contains supplementary material available at 10.1186/s40360-021-00492-z.

## Background

The nonalcoholic fatty liver disease (NAFLD) is increasing worldwide and the most common chronic liver disease. Epidemiological research and animal research suggest that endocrine disruptors are the risk factors that contribute to the metabolic disease such as NAFLD [1]. It is reported that NAFLD is induced by prenatal exposure to bisphenol A as endocrine disruptors in offspring rats [2].

NP as one of the typical environmental endocrine disruptors is a chemical product widely used in life and medicine, which can be released into the environment and enter into the food chain. The main human exposure route is contaminated food and drinking water [3]. The daily per capita intake of NP in Chinese adults is 520 ng/kg. bw/day; The daily per capita intake of NP in children (0 to 6 years old) is 300 ng/kg to 17 μg/kg.bw/day, which is higher than that of adults; In particular, The daily per capita intake of NP in infants and young children is 17 μg/kg.bw/day, which is much higher than the daily tolerable intake (5 μg/kg.bw/day) set by the Danish Environmental Protection Agency [4]. Moreover, NP which is highly lipophilic could enter the fetus through the placental barrier of pregnant women, being harmful to the growth and development of various organs of the fetus [5]. All of them indicate the potential health risks of NP, especially in pregnant women and infants. The prevalence of high-fat and high-sugar diet patterns is also considered to be one of the important factors leading to NAFLD [6]. HFD exposure may promote NP exposure to cause fatty acid synthesis in the liver of the human body, and even lead to NAFLD. Therefore, we speculate that HFD accelerate the synthesis of fatty acids in the liver tissue induced by perinatal exposure to NP.

Early environmental stimuli of life will have a lasting effect on offspring and lead to hereditary diseases of offspring such as obesity and NAFLD. Female rats exposed to an abnormal environment will affect the expression of metabolic phenotypes in offspring rats, which could continue to occur in the next generation or several generations [7, 8]. For example, intrauterine hyperglycemic conditions could impair glucose tolerance and insulin levels in both F1 and F2 generation rats [7]. Low dose of NP exposure and HFD can alter adipose tissue formation [9]. Moreover, adiposity excess alters the placental nutrient transfer and modifies the composition of breast milk, affecting the development and genetic programing of fetal liver, resulting in abnormal fatty acid synthesis [10]. This genetic change, even if which is not exposed to environmental stimuli in the later stages, could be transmitted to the next generation or generations through sperm or eggs, leading to hereditary chronic diseases. Therefore, we established a rat model of early exposure to NP and HFD to study whether HFD accelerate the synthesis of fatty acids in the liver in male offspring rats induced by perinatal exposure to NP.

## Methods

### Animal studies

Healthy wild virgin female and male Wistar rats at 8 weeks of age were purchased from Beijing Vital Rivers Laboratory Animal Technology Co., Ltd. (Beijing, China). The animals were kept under specific pathogen-free conditions in the Experimental Animal Centre of Tongji Medical College and treated according to the Guidelines of the National Institutes of Health for Animal Care and Use. All the procedures were approved by the Ethics Committee of Tongji Medical College, Huazhong University of Science and Technology.

### Maintenance and treatment with nonylphenol and high-fat diet

Virgin female and male Wistar rats with similar body weight, as F0 generation, were housed in conditions of controlled temperature (21–25 °C) at 40–60% relative humidity with alternate day and night. F0 generation rats were treated with phyto-estrogens deficient diet (PEDD, Shanghai Laboratory Animal Center, Shanghai, China; which contains 13.21% fat, 27.18% protein, and 59.61% carbohydrates, with energy of 14.39 kJ/g kcal/g) from the 11 day before being caged to postnatal day (PND) 21, which would exclude the intervention effect by other estrogenic substance in the normal diet. One male rat and two female rats were caged in one cage to obtain the first generation (F1). The gestation day (GD) 0 was defined the time that sperm-positive smear was observed under a microscope. Sixteen pregnant rats were randomly divided into two groups: Control-ND (normal diet) group treated with olive oil (Sigma-Aldrich, St Louis, MO; CAS No. 8001-25-0) and NP-5-ND group treated with NP (5 μg/kg/day), both of which received normal diet. On PND 21, F1 male or female rats with similar body weight were divided into 4 groups: Control-ND group (16 rats) and NP-5-ND group (16 rats), fed with normal diet; Control-HFD group (16 rats) and NP-5-HFD (16 rats), receiving high-fat diet (28.53% fat, 22.33% protein, 49.14% carbohydrates, with energy of 17.36 kJ/g).

**Fig. 1:**
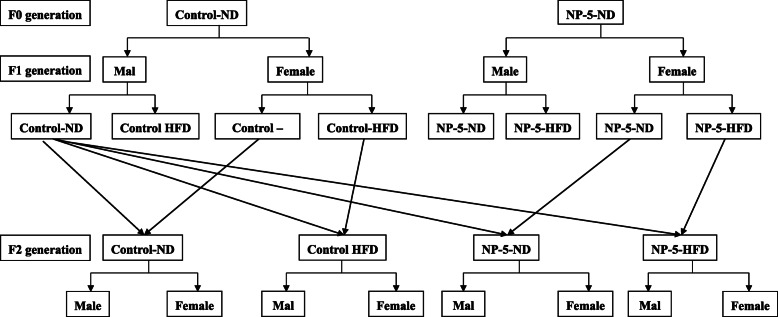
Experimental design and intervention methods

Half of male F1 rats (9 weeks old) in the Control group were mated with half of the female F1 rats (9 weeks old) in the four groups (8 rats per group) to obtain the second generation (F2) rats; the other part of F1 male or female rats were given normal diet or HFD. F2 offspring were all given normal diet after weaning. The experimental design and intervention methods was shown in Fig. 1. F1 male rats and F2 male rats were anesthetized with pentobarbital sodium (intra-peritonealed with 50 mg/kg pentobarbital sodium) and euthanasiaed by cervical dislocation at 23 weeks of age and 13 weeks of age respectively. The serum was collected and stored in a refrigerator at − 80 °C. The liver tissues were separated rapidly, washed by PBS, dried by the filter paper and weighted to figure out the organ coefficient.

### Detection of the level of serum nonylphenol in F1 and F2 male rats

The whole blood which was collected from the PND21 pups in F1 and F2 male rats at the indicated time points after NP exposure was centrifuged to obtain the serum sample. The serum levels of NP were determined with a liquid chromatography tandem-mass spectrometry (LC-MS/MS) according to the method previous reported [10, 11].

### Detection of biochemical indexes in serum of F1 and F2 male rats

The whole blood was collected and centrifuged to obtain the serum. High density lipoprotein (HDL), alanine aminotransferase (ALT), aspartate aminotransferase (AST), total cholesterol (TC) and low density lipoprotein (LDL) were detected by automatic biochemical analyzer (Mindray BS-200; Shenzhen, China).

### Observation of liver histopathology

The liver tissues which were separated on the F1 male rats (23-week and F2 male rats (13-week) were dried by the filter paper after washing by the PBS and put in the 4% paraformaldehyde solution for over 24 h. Firstly, tissue dehydration: the fixed liver tissues were put respectively into different percentage ethanol solutions (30, 50, 70, 80, 90, 95, 100% × 2) every 30 min. Secondly, tissue vitrification: the dehydrated tissue was put successively into ethanol-xylene for 10 min, xylene for 10 min, and xylene II for 10 min. Thirdly, tissue waxing and embedding: the tissues were put into pre-melting 50% wax + 50% xylene for 30 min, then being waxed twice, 2 h for each time and stored under 4 °C as standby. Fourthly, sliced: paraffin-embedded tissue blocks were cut into 5 μm slices via wax cutter. The wax slices were put on the 40 °C water with shiny side downwards, then put on the anti-off slider, being dried 40 °C all over the night. Fifthly, de-waxing: The wax slices were operated with following treatment, xylene for 3–5 min, ethyl alcohol for 3–5 min, 95% ethyl alcohol for 3–5 min, 80% ethyl alcohol for 3–5 min, being washed by water for 2 min double distilled water for 2 min. After Hematoxylin and Eosin staining (HE), the wax slices were sealed with neutral gum and observed under 40× microscope. If the endochylema is red and cell nucleus is blue, it means the dye is successful. The pictures were taken by using a Zeiss Axiocam Camera (Carl Zeiss, Gottingen, Germany) and processed with Photoshop 6.0 (Adobe Systems, Mountain View, CA).

### Real-time reverse transcription polymerase chain reaction (RT-PCR)

Total RNA was isolated from rat fat tissue using the E.Z.N.A. Total RNA Kit (Omega, Norcross, GA, USA) following the manufacturer’s protocol. One-step RT-PCR with real-time detection was conducted with the SYBR Green Real-Time RT-PCR Master Mix (Toyobo, Osaka, Japan). The mRNA levels of *Lpl*, *Fas*, *Srebp-1*, *Ppar-γ*, and *36B4* (Table 1) were detected with the PCR condition: 95 °C,10 min; 95 °C, 15 s, 40 circulations; 60 °C, 1 min. Relative gene expression was calculated by the 2^-△△Ct^ method with 36B4 as an endogenous reference gene.

**Table 1:** PCR Primer Series

Gene name	Gene bank	size (bp)	Primers
*Lpl*	NM_012598.2	169	Forward: CCAGCTGGGCCTAACTTTGA
			Reverse: GGAAAGTGCCTCCATTGGGA
*Fas*	NM_017332.2	195	Forward: GTGGAAGACACTGGCTCGAA
			Reverse: TGGTACACTTTCCCGCTCAC
*Srebp-1*	NM_001276708.1	92	Forward: GGAGCCATGGATTGCACATTT
			Reverse: CCAGCATAGGGGGCATCAAA
*Ppar-γ*	NM_001145367.1	350	Forward: TCAGCTCTGTGGACCTCTCC
			Reverse: ACCCTTGCATCCTTCAGAAG
*ERα*	XM_039101049.1	85	Forward: AAGCCAATCTGTACCTCGGC
			Reverse: CTGGTGCAACAAGGCCATTC
*36B4*	NM_022402.1	141	Forward: CAGCAGGTGTTTGACAATGGC
			Reverse: TGAGGCAACAGTCGGGTAGC

### Western blotting

The liver tissues were lysed in 1 x SDS loading buffer. Aliquots of proteins from tissue lysates were electrophoresed on 10% (w/v) polyacrylamidegels and transferred onto nitrocellulose membranes (Schlei-cher&Schuell, Kassel, Germany). The blot was probed with antibodies for ERα (GB11026, Servicebio Biotechnology Inc., China) or β-actin (GB12001, Servicebio Biotechnology Inc., China) and HRP- conjugated secondary antibodies (GB23404, Servicebio Biotechnology Inc., China) were applied. Then the membranes were washed with PBS with 0.1% Tween 20, incubated in Lumi-Light working solution (GE Health-care, Piscataway, NJ, USA) and exposed to X-ray films.

### Statistical analysis

All data were expressed as mean ± standard error (Mean ± SEM), being analyzed via SPSS 18.0. The comparison between different groups was carried out by using one-way ANOVA and two-way ANOVA with Bonferroni’s test. Comparison between two groups were analyzed by two independent samples t-test. *P* < 0.05 was considered statistically significant.

## Results

### Detection of NP in serum of F1 and F2 male rats

To determine the correlation between the abnormal symptoms of F1 and F2 male rats and the NP exposure in the F0 female rats, the level of serum NP in PND 21 of F1 and F2 male rats was detected in our experiment. The detection limit of liquid phase mass spectrometry was only 0.5 ng/ml. NP was not detected in serum of the F1 and F2 male rats (Table 2).

**Table 2:** Detection of NP concentration in PND21 of F1 and F2 male rats

		F1 (ng/ml)	F2 (ng/ml)
ND	Control	N	N
	NP-5	N	N
HFD	Control	–	N
	NP-5	–	N

Note:*n* = 6 rats/group. —, this group did not exist; N, NP was not detected

### Synergistic effects of HFD with NP on gestation days, litter size, sex ratio and birth weight

F0 and F1 rats during pregnancy did not show significant weight loss, abortion, death and other abnormal situations. NP treated alone (NP-5-ND group) has no significantly change in gestation days, litter size, sex ratio and birth weight in F0 and F1 rats (Table 3). And HFD treated alone has no significant change in gestation days, litter size, sex ratio and birth weight in F1 rats (Table 3). However, the NP and HFD interaction (NP-5-HFD group) had significant synergistic effect on the birth weight of F1 rats (*P* < 0.05), but the other indexes were not changed significantly (Table 3).

**Table 3:** Synergistic effects of HFD and NP on gestation days, litter size, sex ratio and birth weight of F0 and F1 rats

	F0 rats.			F1 rats						
	Control-ND	NP-5-ND	Nonylphenol	Control-ND	NP-5-ND	Control-HFD	NP-5-HFD	Diet	Nonylphenol	D × N
Gestation days	20.53 ± 0.58	21.04 ± 0.27	*P* > 0.05	21.67 ± 0.17	20.32 ± 0.95	20.46 ± 0.78	21.07 ± 1.05	*P* > 0.05	*P* > 0.05	*P* > 0.05
Litter size (mm)	15.00 ± 4.08	13.50 ± 1.00	*P* > 0.05	11.00 ± 1.20	10.57 ± 1.90	10.16 ± 1.11	11.57 ± 2.94	*P* > 0.05	*P* > 0.05	*P* > 0.05
Sex ratio (%)	54.70 ± 6.75	53.85 ± 9.21	*P* > 0.05	56.22 ± 4.25	53.85 ± 9.21	55.85 ± 9.33	54.32 ± 4.51	*P* > 0.05	*P* > 0.05	*P* > 0.05
Birth weight (g)	5.75 ± 0.74	6.07 ± 0.61	*P* > 0.05	5.46 ± 0.14	5.81 ± 0.47	5.41 ± 0.43	6.38 ± 0.65*^#^	*P* > 0.05	*P* > 0.05	*P* < 0.05

Note: Means ± SE. *n* = 6–8 rats/group.*, *P* < 0.05 vs. Control-ND; **, *P* < 0.01 vs. Control-ND; #, Synergistic effect existed vs. Control-ND

### HFD synergistically increased the weight gain and organ coefficient of liver tissue of offspring male rats induced by perinatal exposure to NP

The body weight of F1 (23 weeks old) and F2 (13 weeks old) male rats in NP-5-ND group increased faster than that in Control-ND group (*P* < 0.05; Table 4). The body weight of F1 or F2 male rats in NP-5-HFD group gained faster than that in Control-HFD group (*P* < 0.05; Table 4). Meanwhile, the NP and HFD interaction (NP-5-HFD group) had significant effect on the body weight of male and female rats (*P* < 0.05; Table 4).

**Table 4:** Synergistic effects of HFD and NP on the weight and organ coefficient of liver tissue of F1 and F2 male rats

		ND		HFD		*P*-value		
		Control-ND	NP-5-ND	Control-HFD	NP-5-HFD	Diet	Nonylphenol	D × N
Body weight (g)	F1	384 ± 43	453 ± 52*	417 ± 60	474 ± 69*^#^	< 0.05	< 0.05	< 0.05
	F2	257 ± 25	322 ± 28*	323 ± 24	358 ± 27*^#^	< 0.05	< 0.05	< 0.05
Organ coefficient (%)	F1	2.59 ± 1.92	2.87 ± 2.57*	3.10 ± 0.10	3.53 ± 0.19*^#^	< 0.05	< 0.05	< 0.05
	F2	3.04 ± 0.32	3.16 ± 0.18*	3.02 ± 0.24	3.21 ± 0.55*^#^	> 0.05	> 0.05	> 0.05

Note: *n* = 7–8 rats/group. *, *P* < 0.05 vs. Control-ND; **, *P* < 0.01 vs. Control-ND; #, Synergistic effect existed vs. Control-ND

The organ coefficient of liver tissue in F1 (23 weeks old) male rats in NP-5-ND group increased than that in Control-ND group (*P* < 0.05; Table 4). The organ coefficient of liver tissue in F1 male rats in NP-5-HFD group increased than that in Control-HFD group (*P* < 0.05; Table 4). Meanwhile, the NP and HFD interaction (NP-5-HFD group) had significant effect on organ coefficient of liver tissue in F1 male rats (*P* < 0.05; Table 4). However, there was no significant difference in the data of organ coefficient of the liver tissue in F2 (13 weeks old) rats (Table 4).

### HFD synergistically aggravated the damage of liver function of F1 and F2 male rats induced by perinatal exposure to NP

NP treated alone (NP-5-ND group) up-regulated the levels of the AST in F1 male rats and ALT, TC, LDL of F2 male rats, and down-regulated the levels of HDL of F2 male rats, which had statistical significance (*P* < 0.05, Table 5); While there was no significant difference in the data of biochemical parameters in HFD treated alone of F1 and F2 male rats (*P* > 0.05, Table 5). The NP and HFD interaction (NP-5-HFD group) had significant effect on the level of AST, ALT, TG, TC, LDL of F1 and F2 male rats, which had statistical significance (*P* < 0.05 or *P* < 0.01, Table 5).

**Table 5:** The Combined Effect of HFD and NP Exposure on the Blood Biochemical Parameters in F1 and F2 male Rats

		ND		HFD		*P*-value		
		Control-ND	NP-5-ND	Control-HFD	NP-5-HFD	Diet	Nonylphenol	D × N
ALT (U/L)	F1	85.05 ± 15.9	64.96 ± 12.93	97.17 ± 7.56	115.75 ± 7.05*^#^	> 0.05	> 0.05	< 0.05
	F2	57.84 ± 6.10	72.57 ± 5.81*	59.98 ± 2.60	80.16 ± 5.52*^#^	> 0.05	< 0.05	< 0.05
AST (U/L)	F1	181 ± 13	212 ± 5*	201.94 ± 15.73	240 ± 23.53*^#^	> 0.05	< 0.05	< 0.05
	F2	237 ± 16	235 ± 7	251.42 ± 21.97	296.34 ± 8.19*^#^	> 0.05	> 0.05	< 0.05
TG (mmol/L)	F1	0.68 ± 0.15	0.52 ± 0.09	1.23 ± 0.03	1.61 ± 0.04*^#^	> 0.05	> 0.05	< 0.05
	F2	0.72 ± 0.21	0.78 ± 0.11	0.93 ± 0.20	1.70 ± 0.15*^#^	> 0.05	> 0.05	< 0.01
TC (mmol/L)	F1	1.97 ± 0.28	2.07 ± 0.08	2.15 ± 0.46	2.54 ± 0.29*^#^	> 0.05	> 0.05	< 0.05
	F2	1.97 ± 0.21	3.38 ± 0.32*	2.09 ± 0.09	3.63 ± 0.08*^#^	> 0.05	< 0.05	< 0.01
HDL (mmol/L)	F1	2.06 ± 0.28	1.81 ± 0.09*	2.24 ± 0.37	2.18 ± 0.33	> 0.05	< 0.05	> 0.05
	F2	1.88 ± 0.06	1.60 ± 0.13*	2.03 ± 0.12	1.75 ± 0.14	> 0.05	< 0.05	> 0.05
LDL (μg/mL)	F1	0.25 ± 0.06	0.30 ± 0.04*	0.29 ± 0.08	0.39 ± 0.04*^#^	> 0.05	< 0.05	< 0.05
	F2	0.29 ± 0.01	0.57 ± 0.01	0.29 ± 0.05	0.68 ± 0.12*^#^	> 0.05	< 0.05	< 0.01

Note: Means ± SE. *n* = 8 rats/group. *, *P* < 0.05 vs. Control-ND; **, *P* < 0.01 vs. Control-ND; #, Synergistic effect existed vs. Control-ND

### HFD aggravated the damage of liver tissue of F1 and F2 male rats induced by perinatal exposure to NP

The liver tissues were cut and observed by naked eyes. In the Control-ND group, NP-5-ND group and Control-HFD group, the livers of two generation rats were bright red with sharp edges and smooth surfaces. But livers in the NP-5-HFD groups of F1 and F2 rats were blunt with yellowish brown color edges.

**Fig. 2:**
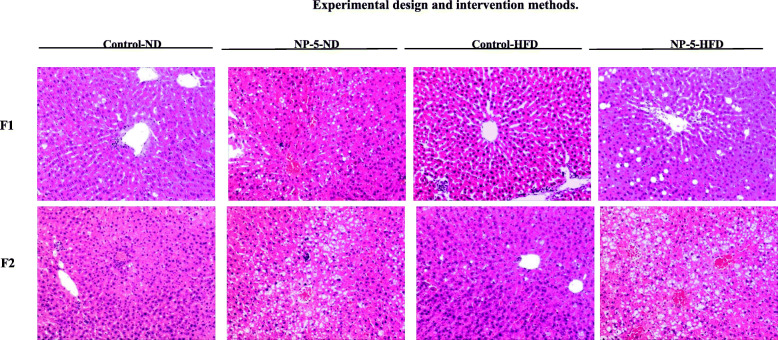
The results of histopathologic staining in the liver tissues on F1 and F2 male rats (40×). Compared to the Control-ND group, dilated hepatic sinus and scattered disorderly liver cells were found in NP-5-ND group and Control-HFD group. NP-5-ND group had incomplete structure of some hepatic lobule and vacuoles even the lipid droplets. NP-5-HFD group also had incomplete structure of some hepatic lobule with unclear edge of the pipe area and more severe vacuoles or lipid droplets formation. Note: * indicate *P* < 0.05; ** indicate *P* < 0.01

It showed that the round central nucleus and the abundant cytoplasm of liver cell were arranged radially from the central vein of the liver in HE staining in the Control-ND group of F1 and F2 male rats. There were some complete hepatic lobules and portal areas with no dilatation of the hepatic sinus. Compared to the Control-ND group, dilated hepatic sinus and scattered disorderly liver cells were found in NP-5-ND group and Control-HFD group in F1 and F2 male rats. NP-5-ND group in F1 and F2 male rats had incomplete structure of some hepatic lobule and vacuoles even the lipid droplets. NP-5-HFD group in F1 and F2 male rats also had incomplete structure of some hepatic lobule with unclear edge of the pipe area and more severe vacuoles or lipid droplets formation (Fig. 2).

### HFD synergistically up-regulated the fatty acid synthesis gene of the liver of F1 and F2 male rats induced by perinatal exposure to NP

**Fig. 3:**
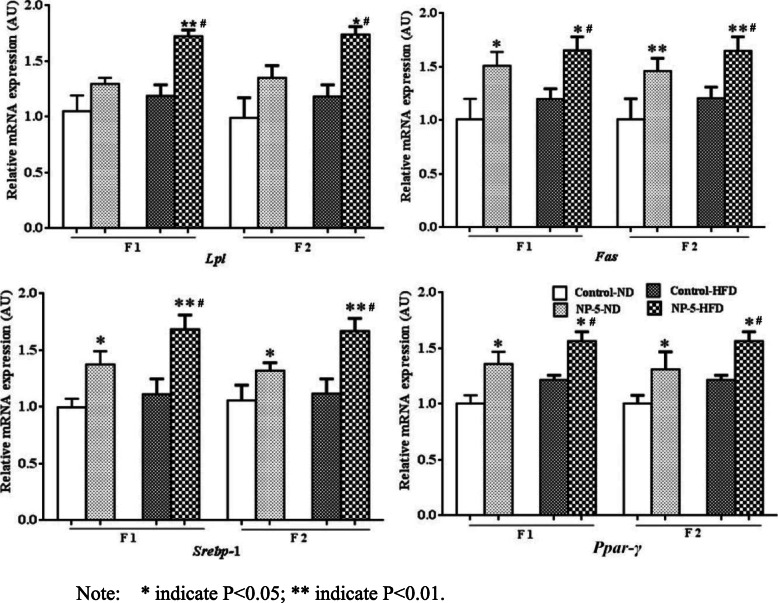
HFD synergistically up-regulated the fatty acid synthesis gene of the liver of F1 and F2 male rats induced by perinatal exposure to NP. The NP treated alone rats significantly up-regulated the mRNA levels of Fas, Srebp-1, Ppar-γof F1 and F2 male rats. The NP and HFD interaction had significant synergistic effect on the mRNA levels of Lpl, Fas, Srebp-1, Ppar-γ in F1 and F2 male rats. *, *P* < 0.05 vs. Control-ND; **, *P* < 0.01 vs. Control-ND; #, Synergistic effect existed vs. Control-ND

To further confirm whether HFD aggravated the synthesis of fatty acids in the liver of male offspring rats induced by NP, we detected the mRNA levels of the fatty acid synthesis in the liver tissue, such as Lipoprotein lipase (*Lpl*), Fatty acid synthetase (*Fas*), sterol regulatory element-binding protein 1 (*Srebp-1*), and Peroxisome proliferator activated receptor γ (*Ppar-γ*). The NP treated alone rats (NP-5-ND group) significantly up-regulated the mRNA levels of *Fas*, *Srebp-1*, *Ppar-γ* of F1 and F2 male rats (*P* < 0.05 or *P* < 0.01, see Fig. 3). While there was no significant difference in the data of fatty acid synthesis gene in HFD treated alone of F1 and F2 male rats. The NP and HFD interaction (NP-5-HFD group) had significant synergistical effect on the mRNA levels of *Lpl*, *Fas*, *Srebp-1*, P*par-γ* of F1 and F2 male rats (*P* < 0.05 or *P* < 0.01, see Fig. 3).

### Synergistical effects of NP and HFD on the expression of ERα in the liver tissues of F2 male rats

**Fig. 4:**
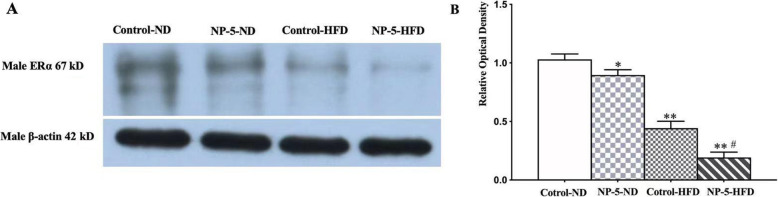
Synergistic effects of HFD and NP on the expression of ERα in the liver tissue in F2 male rats. NP or HFD alone significantly decreased the expression level of ERα in F2 male rats respectively. The NP and HFD interaction synergistically decreased the expression of ERα in liver tissue of F2 male rats. Note: *, *P* < 0.05 vs. Control-ND; **, *P* < 0.01 vs. Control-ND; #, Synergistic effect existed vs. Control-ND. The full-length blots/gels are presented in Supplementary Figure 1

Recent reports have demonstrated that NP could bind to the estrogen receptors (ERs) and promote excessive accumulation of fatty acid. The NP and HFD interaction had significant synergistical effect on the mRNA levels of fatty acid synthesis gene in F1 and F2 male rats, raised the question that whether ER played a vital role in the synergistic effect of NP and HFD on induction of liver fatty acid synthesis in F2 male rats. We detected the protein expression of ERα by western blotting (Fig. 4). NP or HFD alone significantly decreased the protein expression level of ERα of liver tissue in F2 male rats respectively. The NP and HFD interaction synergistically decreased the protein expression of ERα in liver tissue of F2 male rats (Fig. 4).

## Discussion

The early environmental stimuli of life will have a lasting impact on future generations, even lead to hereditary diseases of the offspring [8, 9]. We mainly studied that the HFD accelerated the synthesis of fatty acids in the liver of F1 and F2 adult rats induced by perinatal exposure to NP. Liver is the main organ in lipid metabolism. Fatty acids and glycerol synthesize triglycerides in the liver, then triglycerides synthesize into very low density lipoproteins (VLDL) by combining with apolipoproteins, cholesterol, etc., which are transported to the extrahepatic tissues for storage or utilization. If the liver’s lipid metabolism is impaired, hepatic steatosis will form [12]. To determine the association between abnormal feature in F1 and F2 rats and NP (5 μg/kg/day) exposure in F0 female rats, we confirmed that levels of the serum NP in postnatal day 21 of F1 and F2 rats was zero (Table 2). In addition, the HFD was consisted of 28.53% fat, 22.33% protein and 49.14% carbohydrate, and the energy was 17.36 kJ/g. Although NP or HFD treated alone has no significantly changes in gestation days, litter size, sex ratio and birth weight in offspring rats, the NP and HFD interaction had significant synergistic effect on the birth weight of F1 rats. The body weight and the organ coefficient of liver tissue of offspring male rats in NP or HFD treated alone group increased faster than that in control group. And the NP and HFD interaction had significant synergistic increasing effect on the body weight and the organ coefficient of liver tissue. In another words, HFD synergistically stimulated increasing of the birth weight, body weight and liver tissue organ coefficient of offspring rats induced by perinatal exposure to nonylphenol. Our results also indicated that NP treated alone increased the abnormal levels of biochemical indexes of male offspring rats while there was no significant difference in data of HFD treated alone group. And HFD synergistically aggravated the damage of liver function and abnormality of blood lipid in offspring male rats induced by perinatal exposure to NP. NP or HFD treated alone group showed the significant damage of liver tissue of F1 and F2 male rats. And HFD also synergistically aggravated the damage of liver tissue of F1 and F2 male rats induced by perinatal exposure to NP showed in NP-5-HFD group. In another words, HFD synergistically aggravated abnormal lipid metabolism, even liver function and liver histopathological damage of offspring male rats for perinatal exposure to NP, which showed genetical changes and could pass on the next generation rats.

The liver is the most active organ for lipid metabolism in the body. Many genes are involved in liver lipid metabolism, such as *Lpl*, *Fas*, *Srebp-1*, *Ppar-γ*, which are well known and are mainly located in the cytoplasm of liver cells. *Lpl* is a critical enzyme in the lipid metabolism, which provide fatty acids and monoacylglycerol by catalyses the hydrolysis of the triacylglycerol component of chylomicrons and very low density lipoproteins [13]. *Fas* is one of the key enzymes for fatty acid synthesis, which Catalyzes the conversion of acetyl-CoA and malonyl-CoA to fatty acids required for human fat deposition. Increasing *Fas* gene expression can promote fat synthesis, whereas reducing *Fas* gene expression can reduce fat synthesis and achieve the purpose of preventing and treating fatty liver [14]. *Srebp-1* is an important transcriptional regulator that regulates lipid synthesis. *Srebp-1* exists in the endoplasmic reticulum as an inactive precursor. Activated *Srebp-1* enters the nucleus and induces the expression of genes related to lipid synthesis [15]. *Ppar-γ* belong to the nuclear receptor superfamily, which regulating the lipid homeostasis [16]. Our results suggested that the 4-NP treated alone rats significantly up-regulated the mRNA levels of *Lpl*, *Fas* and *Srebp-1* of F1 and F2 rats. Among them, the *Srebp-1* is a key gene regulating fat synthesis in the liver, which can induce the synthesis of acetyl-CoA carboxylase (*ACC*) and *Fas*. Studies have shown that long-term intake of HFD increases the expression levels of genes related to fat synthesis in the liver, such as *Srebp-1*, *ACC,* and *Fas*, while decreases the expression levels of genes related to fat decomposition, such as *Ppar-γ*, significantly increases hepatic total fat, triacylglycerides and free fatty acids content [17–19]. HFD produces a significant alteration in the synthesis of fatty acids: an increase in the synthesis of saturated fatty acids (specifically of palmitic acid) via an increase in the activity of FAS and ACC, and a decrease of the hepatic synthesis of polyunsaturated fatty acid [20, 21]. Palmitic acid is the main component of animal and plant fats. The intake of HFD increases the content of palmitic acid in the blood of rats. Studies have shown that palmitic acid can promote the secretion of inflammatory factors, such as *TNF*-α, *IL*-1β and *IL*-6, cause inflammation of liver cells and promote the occurrence of NAFLD [22]. Fatty acids is not only a molecule that provides energy, but also a metabolic regulator. They can act as signaling molecules, directly affecting intracellular or extracellular sensor systems, or when converted into specific fatty acid derivatives. An example of such a sensor system is PPARs. When fat intake is excessive, the expression of *Ppar*-α in the liver is significantly decreased, while the expression of *Ppar*-γ (a transcription factor that controls hepatic FA β-oxidation) is increased, thereby promoting lipogenesis [23]. In addition, it has been showed that in mouse models of insulin resistance, genes implicated in sugar metabolism are down-regulated by the normal activation of genes encoding the lipogenic transcription factor *Srebp-1* [24]. Excess sugar compounds are converted to fat and stored in tissues such as the liver and fat. So the NP and HFD interaction had significant effect on the mRNA levels of *Lpl*, *Fas*, *Srebp-1*, *Ppar-γ* of F1 and F2 male rats. In another words, HFD synergistically accelerated the expression of fatty acid synthesis gene such as *Lpl*, *Fas*, *Srebp-1* and *Ppar-γ* of offspring male rats induced by perinatal exposure to NP, which may have genetic changes and could pass on to the next generation rats. NP and HFD interaction had synergistically aggravated abnormal lipid metabolism and even abnormal liver function and hepatic steatosis that is closely related to up-regulation of fatty acid synthesis gene and excessive accumulation of fatty acids in the liver of male rats, which is consistent with the toxicity of other environmental endocrine disruptors [25]. At the same time, the pro-inflammatory state of F1 pregnancy rats altered placental fatty acid transporters, leading to the imbalance of nutrient transport, including fatty acids. In addition, the mother’s diet, hormonal and metabolic changes also affect breast feeding and milk composition. In general, NAFLD causes changes in the levels of polyunsaturated fatty acids in breast milk and fetal circulation, decreases in *n*-3 polyunsaturated fatty acids (mainly eicosapentaenoic acid and docosahexaenoic acid), and increases in *n*-6 polyunsaturated fatty acids (mainly Linoleic acid and Arachidonic acid). These associations can persist throughout a child’s life and exacerbate abnormal fatty acid synthesis in the liver [10, 26]. More importantly, such excessive accumulation of fatty acids that NP and HFD interaction had synergistically aggravated could pass on to the next generation rats through the germ cells.

The mechanism of environmental factors leading to liver steatosis in genetic changes is still not completely clear. Current research showed that one of main factor which cause disorder of lipid metabolism was closely related to abnormal estrogen receptor, which may increase the risk of liver lipid metabolism in offspring rats. Clinical evidence shows that postmenopausal women may easily obtain weight gain, but estrogen replacement therapy mitigated this phenomenon even in the presence of increased abdominal fat [27]. ERα is one of the estrogen receptors. There was increased adipose tissue in ERα knockout mice [28]. The change of ERα in offspring rats produced by HFD and NP, which regulate mechanism for lipid deposition, is also full of controversial and complicated [29, 30]. Our study demonstrated that NP reduced the protein expression level of ERα in liver tissue of F2 male rats respectively. NP and HFD interaction resulted in synergistical decrease of the protein expression of ERα in liver tissue in F2 male rats, which produced excessive accumulation of fatty acid by altering transcription of fatty acid synthesis gene. More terribly, decrease of ERα may produce genetic effect in liver steatosis of offspring mice. ERα regulates mitochondrial function and energy homeostasis via coordinated control of mtDNA replication by Polg1 and fission- fusion-mitophagy dynamics [31, 32]. Mitochondrial dysfunction and its resulting reduction of mitochondrial energy reserve and enhancement of oxidative stress lead to decreased response ability of steatotic hepatocytes and thus play an important role in the occurrence and development of fatty liver disease.

## Conclusion

HFD and NP synergistically accelerated synthesis of fatty acids in liver of male offspring rats by reducing the expression of ERα, altered transcription of fatty acid synthesis gene and induced abnormal lipid metabolism, abnormal liver function and hepatic steatosis. Moreover, all of these damages passed on to the next generation rats. In-depth studies on the genetic variation related with fat synthesis and decomposition will help reveal the mechanisms. In terms of chronic disease susceptibility, reduced ERα effects impair mitochondrial function, promote obesity, and disrupt metabolic homeostasis in mice and humans. Thus, the role of ERα in liver tissue may be an attractive therapeutic target for the fight against fatty liver and metabolic dysfunction, especially in women during menopausal transition.

## Supplementary Information

**Additional file 1: Supplementary Figure 1.** The full-length blots of ERα in the liver tissue in F2 male. rats.

## Data Availability

All data generated or analyzed during this study are included in this published article .

## Abbreviations

NP: Nonylphenol
NAFLD: Non-alcoholic fatty liver disease
NP-5: Nonylphenol (5 μg/kg/day)
HFD: High-fat diet
ND: Normal diet
TG: Triglyceride
TC: Total cholesterol
LDL: Low density lipoprotein
HDL: High density lipoprotein
ALT: Alanine aminotransferase
AST: Aspartate aminotransferase
VLDL: Very low density lipoproteins
*Lpl*: Lipoprotein lipase
*Fas*: Fatty acid synthetase
*Srebp-1*: Sterol regulatory element-binding protein 1
*Ppar-γ*: Peroxisome proliferator activated receptor γ
ERs: Estrogen receptors
PND: Postnatal day
GD: Gestation day
LC-MS/MS: Liquid chromatography tandem-mass spectrometry
HE: Hematoxylin and eosin staining
RNA: Ribo nucleic acid
RT-PCR: Real-time reverse transcription polymerase chain reaction
