# Supplementary figures and images for: High-fat diet accelerate hepatic fatty acids synthesis in offspring male rats induced by perinatal exposure to nonylphenol
Source: BMC Pharmacol Toxicol. 2021 Apr 27;22:22. doi: 10.1186/s40360-021-00492-z (PMC8077693; doi:10.1186/s40360-021-00492-z)

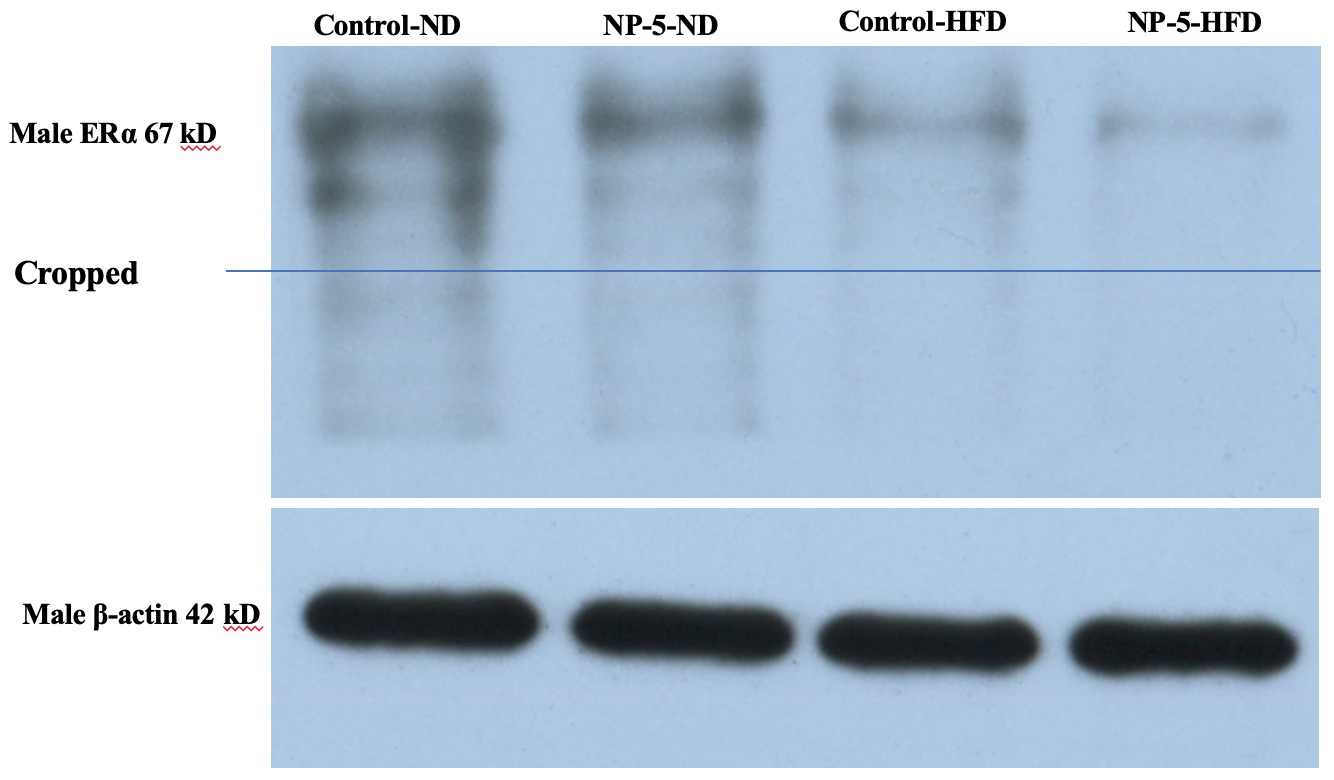


**Supplementary Figure 1. full-length blots of ERα in the liver tissue in F2 male. rats**

Supplement: Supplementary file 1 — Additional file 1: Supplementary Figure 1. The full-length blots of ERα in the liver tissue in F2 male. rats. [file 40360_2021_492_MOESM1_ESM.docx]
